# Structural insights into the inhibitory mechanism of angiotensin‐I‐converting enzyme by the lactotripeptides IPP and VPP

**DOI:** 10.1002/1873-3468.14768

**Published:** 2023-11-03

**Authors:** Kyle S. Gregory, Gyles E. Cozier, Sylva L. U. Schwager, Edward D. Sturrock, K. Ravi Acharya

**Affiliations:** ^1^ Department of Life Sciences University of Bath UK; ^2^ Department of Integrative Biomedical Sciences Institute of Infectious Disease and Molecular Medicine, University of Cape Town South Africa

**Keywords:** angiotensin‐1‐converting enzyme, domain‐selectivity, enzyme structure, inhibitor binding, metalloprotease, X‐ray crystallography

## Abstract

Human somatic angiotensin‐1‐converting enzyme (sACE) is composed of a catalytic N‐(nACE) and C‐domain (cACE) of similar size with different substrate specificities. It is involved in the regulation of blood pressure by converting angiotensin I to the vasoconstrictor angiotensin II and has been a major focus in the development of therapeutics for hypertension. Bioactive peptides from various sources, including milk, have been identified as natural ACE inhibitors. We report the structural basis for the role of two lacototripeptides, Val‐Pro‐Pro and Ile‐Pro‐Pro, in domain‐specific inhibition of ACE using X‐ray crystallography and kinetic analysis. The lactotripeptides have preference for nACE due to altered polar interactions distal to the catalytic zinc ion. Elucidating the mechanism of binding and domain selectivity of these peptides also provides important insights into the functional roles of ACE.

## Graphical abstract

The milk‐derived lactotripeptides LPP and VPP have shown promising results in the reduction of hypertension by inhibiting the N‐ and C‐domain (nACE and cACE respectively) of angiotensin‐1‐converting enzyme. This study reveals the molecular interactions that underpin this inhibition by the resolution of two X‐ray crystal structures (nACE:IPP and nACE:VPP). Additionally, we present the kinetic parameters of inhibition. Both IPP and VPP bind nACE in the same manner, however, IPP provides stronger inhibition.

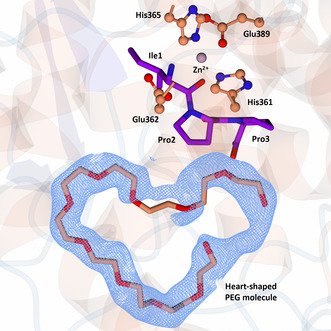

## Abbreviations

**ACE**, angiotensin‐1‐converting enzyme

**Ang I**, angiotensin I

**Ang II**, angiotensin II

**cACE**, angiotensin‐1‐converting enzyme C‐domain

**nACE**, angiotensin‐1‐converting enzyme N‐domain

**RAAS**, renin–angiotensin–aldosterone system

**sACE**, somatic angiotensin‐1‐converting enzyme

**tACE**, testis angiotensin‐1‐converting enzyme

Human angiotensin‐I‐converting enzyme (ACE, EC3.4.15.1) is a zinc‐dependent dipeptidyl carboxypeptidase involved in the regulation of blood pressure *via* the renin–angiotensin–aldosterone and kallikrein–kinin systems. ACE functions by activating angiotensin‐I to the potent vasoconstrictor angiotensin II, and through the metabolic inactivation of the vasodilatory peptide bradykinin [1, 2, 3, 4]. ACE is a type I transmembrane glycoprotein consisting of a short C‐terminal cytoplasmic domain, a transmembrane region and a large extracellular N‐terminal catalytic domain. There are two isoforms, somatic ACE (sACE) and testicular ACE (tACE), with the catalytic domain of sACE made up of two homologous domains (N‐domain, nACE and C‐domain, cACE) that share 60% overall sequence identity and 89% similarity [5, 6]) that are connected by a short flexible linker. tACE is expressed solely in the testis [7], whereas sACE is expressed in epithelial, endothelial, neuroepithelial and immune cells [8]. Despite the high sequence and structural similarity of the active sites of the nACE and cACE domains, they possess differences in substrate specificities, catalytic efficiencies, thermal stabilities and chloride‐ion dependences [9, 10, 11, 12]. The different substrates recognised and cleaved by sACE include angiotensin‐I [13, 14], *N*‐acetyl‐seryl‐aspartyl‐lysyl‐proline (Ac‐SDKP) [15], bradykinin [16], substance P [17], enkephalins [18], luteinising hormone‐releasing hormone [19], neurotensin [20] and amyloid‐beta [21]. Although nACE and cACE possess a similar specificity for angiotensin I, cACE has a 3‐fold higher turnover of angiotensin I to angiotensin II [22]. Therefore, cACE is the major contributor to blood pressure regulation through hydrolysis of Ang I and bradykinin [23, 24], while nACE is predominantly involved in haematopoietic stem cell [25] and fibroblast proliferation [26] through the hydrolysis of Ac‐SDKP. The currently available clinical inhibitors of sACE bind both nACE and cACE with limited domain‐specificity, leading to side effects such as a dry cough and angioedema. These side effects are thought to arise due to the upregulation of bradykinin levels following sACE inhibition [27]. Bradykinin is a non‐selective sACE substrate cleaved by both domains with similar catalytic efficiencies [16, 28], and as part of the kallikrein–kinin system, acts to increase vascular permeability and dilation [29]. This illustrates the need for the design of domain‐specific inhibitors, that can inhibit the catalytic activity of cACE or nACE without disrupting the other domain.

The first identified ACE inhibitors, bradykinin‐potentiating peptides (BPPa, b, c and 2), were isolated from Brazilian pit viper venom with BPPa, BPPb and BPP2 possessing selectivity towards cACE. These inhibitors have an Ile‐Pro‐Pro (IPP) motif at their C terminus and were used to guide the structure‐based design of the widely utilised synthetic ACE inhibitor captopril [30]. There has also been interest in the development of ‘functional foods’ for the treatment and prevention of cardiovascular diseases in a nonpharmacological manner [31]. The lactotripeptides Ile‐Pro‐Pro (IPP) and Val‐Pro‐Pro (VPP) isolated from fermented milk and casein hydrolysates provide vasodilatory affects through the inhibition of sACE, with IC_50_ values for sACE of 5 and 9 μm [32] respectively. Furthermore, they have shown promising results in both animal models and clinical trials for the reduction of blood pressure with no adverse events [31, 33, 34, 35]. One study showed that fermented milk products have enhanced hypotensive activity compared to isolated IPP and VPP [33] suggesting that additional components of milk contribute to the observed reduction in blood pressure. Although IPP and VPP have a relatively weak affinity for sACE (compared to other ACE inhibitors) [36], they appear to be effective for the treatment of hypertension. Indeed, this is a relatively ‘new’ strategy where the design of promiscuous weak drugs for the treatment of complicated disease pathologies such as cancers and cardiovascular disease [37], limits the prevalence of adverse events and encourages off‐target binding with potential beneficial effects.

Determining the precise mechanism of IPP and VPP binding, and their subtle selectivity will support their potential use as therapeutics and also provide insights into N‐ and C‐domain selectivity. Furthermore, the presence of *cis* or *trans* configuration IPP and VPP tripeptides may influence their bioactivity. Based on *in silico* modelling, the *cis* configuration of the first peptide bond was suggested to be more potent than the *trans* configuration [38]. Here we present a detailed kinetic characterisation of nACE and cACE inhibition by synthetic *trans‐*IPP and *trans*‐VPP along with high‐resolution crystal structures of nACE in complex with these two tripeptides. A comparison of the kinetic data presented here for the synthetic *trans*‐tripeptides to the kinetic data from tripeptides isolated from milk obtained previously [12], and the crystal structure of nACE in complex with *trans*‐IPP and *trans*‐VPP indicate that there may be preferential binding for *trans*‐IPP/VPP with selectivity towards nACE. These findings will illuminate the impact of the *trans* configuration of these tripeptides on their bioactivity, which could have important implications for the manufacture of functional foods to treat hypertension and clarify their binding kinetics.

## Materials and methods

### Protein expression and purification

Minimally glycosylated and truncated N‐domain (N389, nACE) and C‐domain (g13, cACE) (Ser‐1 to Pro‐633) human ACE were expressed in cultured mammalian CHO cells and purified as previously described [39, 40].

### Tripeptides

Both IPP and VPP tripeptides used in the present study (in *trans* configuration) were synthesised by GL Biochem (Shanghai, China) for crystallographic studies, and Phoenix Pharmaceuticals, Inc. (Burlingame, CA, USA) for kinetic characterisation of IPP and VPP binding.

### Kinetic characterisation of IPP and VPP binding

Ile‐Pro‐Pro and VPP binding affinity with nACE and cACE were determined using a modified Z‐phenyl‐alanyl‐histidyl‐leucine (Z‐FHL) (Bachem, Bubendorf, Switzerland). IPP and VPP were dissolved in water and a serial dilution prepared in 100 mm potassium phosphate buffer, pH 8.3, containing 300 mm NaCl and 10 μm ZnSO_4_. Equal volumes of enzyme and IPP/VPP were mixed and incubated at room temperature for 40 min. 25 μL of the reaction was aliquoted onto a 96‐well plate in triplicate. 25 μL of Z‐FHL at 1 mm was added to each well, incubated at 37 °C for 15 min and the remainder of the assay was performed as previously described [41]. Initial reaction velocities were analysed using nonlinear regression in prism – graphpad 9.0.2 to obtain IC_50_ values. Domain selectivity factors for IPP and VPP were calculated using IC_50_(C)/IC_50_(N), for comparison to previously reported selectivity factors.

### X‐ray crystallography

Angiotensin‐1‐converting enzyme N‐domain was concentrated to 5 mg·mL^−1^ and mixed with equal volumes of 20 mm IPP/VPP dissolved in water. The complex was left to equilibrate at room temperature for ~ 1 h prior to setting up crystallisation. nACE‐IPP and nACE‐VPP crystals formed by hanging‐drop vapour diffusion at 16 °C in a 1 : 1 μL ratio of protein‐tripeptide complex with 30% PEG 550 MME/PEG 20000, 0.1 m Tris/Bicine pH 8.5 and 60 mm divalent cations [Molecular Dimensions (Rotherham, UK) Morpheus A9]. Crystals were mounted onto a cryoloop and flash frozen in liquid nitrogen for X‐ray diffraction data collection at 100 K. A total of 7200 images were taken at 0.1° of oscillation with an exposure time of 0.05 s per image. Raw images were indexed and integrated using DIALS [42], with subsequent data processing performed using the CCP4 suite [43], including data reduction with AIMLESS, phase estimation with Phaser [44] (using 6F9V [45] as the model for molecular replacement), and refinement with REFMAC5 [46] and Coot [47]. The tripeptides IPP and VPP, zinc ions, chloride ions and purification/crystallisation buffer reagents were added based on the dFo‐mFc Fourier difference map. The structures were validated using Molprobity [48] and figures created with CCP4MG [49].

## Results and Discussion

### Crystal structure of nACE‐IPP and nACE‐VPP peptide complexes

**Fig. 1:**
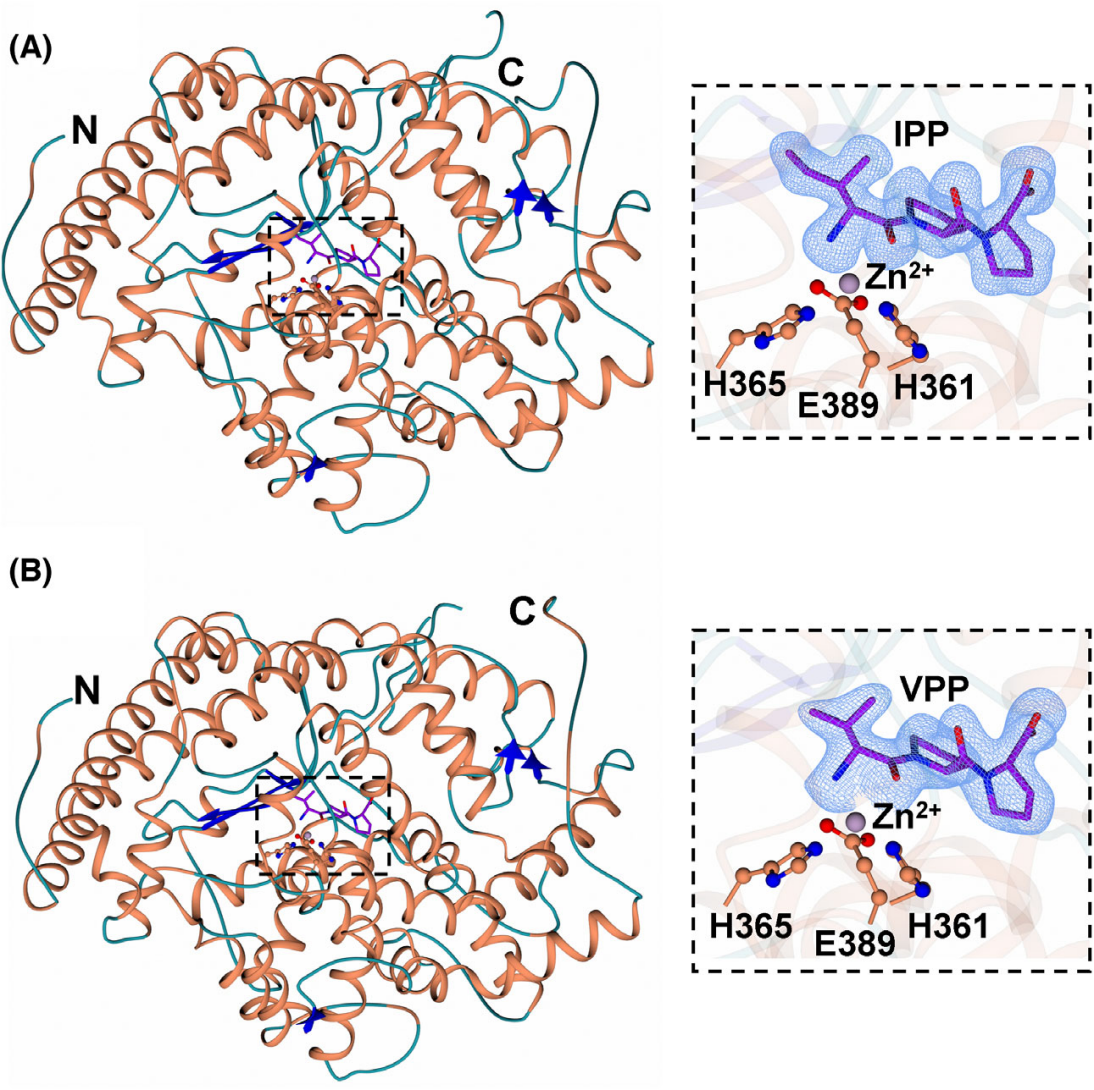
Schematic representation of the overall nACE‐IPP (A) and nACE‐VPP (B) peptide complex structures. nACE side chain residues and α‐helices are shown in orange, β‐strands in blue and loops in light blue. Zinc ions are shown as lilac spheres. IPP and VPP are show in purple. Insert shows the F_O_‐FC omit map (in blue contoured to 3 σ) for which the tripeptides could be modelled. Figure was generated using CCP4MG.

**Fig. 2:**
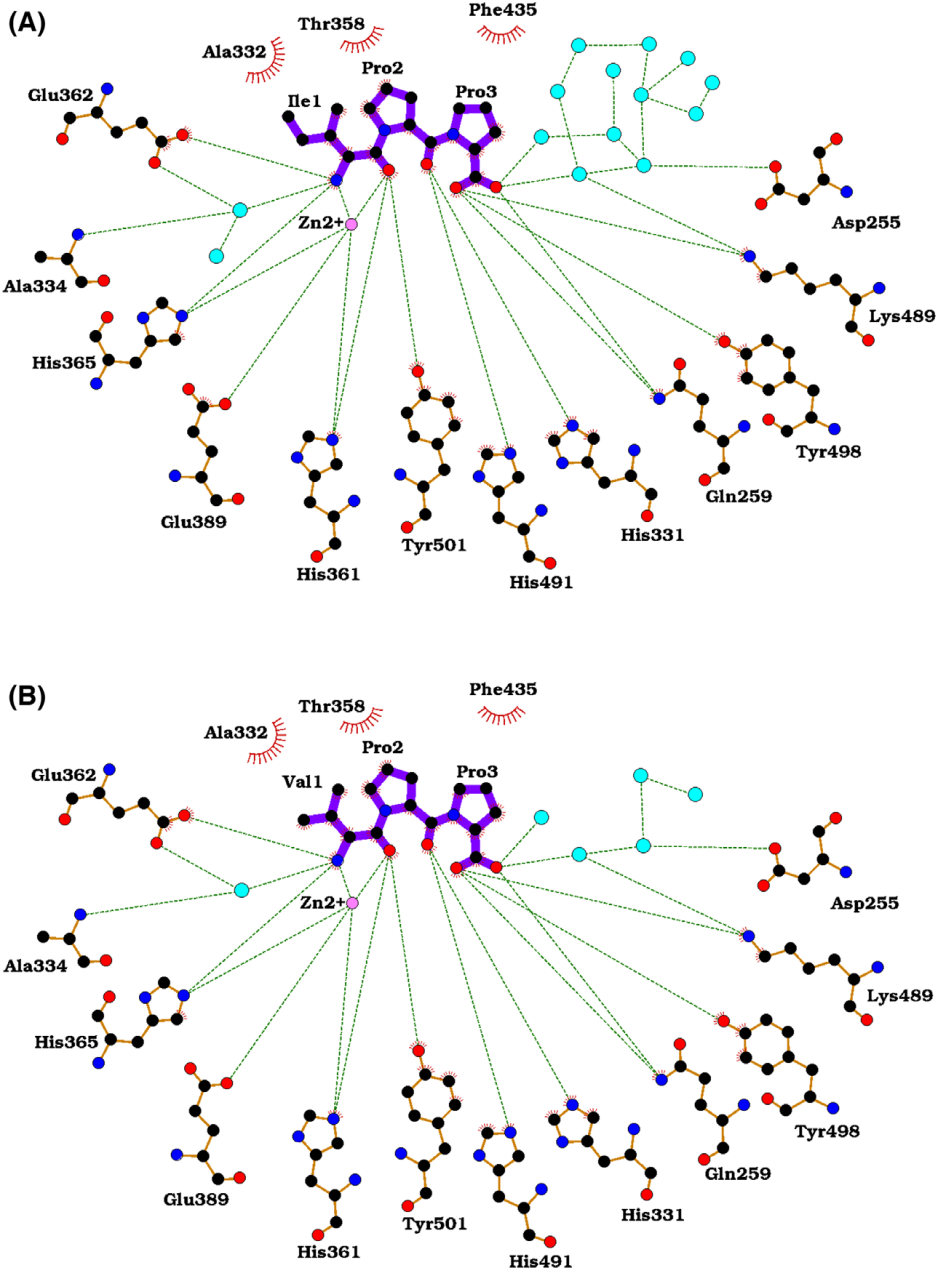
Ligplot representation of nACE‐IPP (A) and nACE‐VPP (B) peptide complexes. The nACE‐VPP peptide complex interactions are identical to the interactions observed for nACE‐IPP peptide complex. H‐bond/electrostatic interactions are shown by green dashed lines. Hydrophobic contacts are shown by the red semi‐circular symbols. Figure was generated using Ligplot+.

The crystal structures of nACE‐IPP and nACE‐VPP peptide complexes were determined at high resolution (1.60 and 1.90 Å respectively) in the P1 space group with unit cell dimensions characteristic of the previously determined nACE ‘closed’ structures (Table 1). Both structures display the typical two lobed α‐helical ellipsoidal fold (Fig. 1A,B), which is thought to open and close to grant substrates access to the active site [50]. Superimposition of nACE‐IPP and nACE‐VPP results in an RMSD value of 0.48 Å (for 600 Cα atoms of molecule A) indicating high structural similarity. Inspection of the mFo‐DFc electron density maps of nACE‐IPP and nACE‐VPP complexes close to the active site revealed clear electron density, for which IPP and VPP could be modelled (Fig. 1A,B, inset). IPP and VPP adopt a *trans* configuration across all three amino acids, with the N‐terminal Ile1 or Val1 residue occupying the S_1_ subsite and Pro2 and Pro3 occupying the S_1_′ and S_2_′ subsites, respectively. Pro2 is sandwiched between His331 and His361, and Pro3 forms a π‐stacking interaction with Tyr501. The tripeptides coordinate the active site directly, *via* both the N‐terminal amino group and backbone carbonyl, effectively straddling the Zn^2+^ ion. This indicates a potential pH dependency of the interaction, given that the terminal amino group must be deprotonated in order to be coordinated. Additionally, the N‐terminal amino group forms water‐mediated interactions with Glu362, and the peptide backbone of Ala334. The carbonyl of Pro2 is coordinated by His331 and His491, and the carboxyl group of Pro3 by Tyr498, Lys489, Gln259 and a network of water‐mediated interactions across the active site cavity (Fig. 2) which may act to neutralise the repulsion of the terminal carboxylic acid group by Asp255. For clarity, as the crystal structures of nACE‐IPP and nACE‐VPP complexes are identical in terms of amino acid side chain positioning, tripeptide binding mode and interacting residues, all further structural analyses reference the higher resolution nACE‐IPP complex structure.

**Table 1:** X‐ray data collection and refinement statistics. Data from the inner shell are shown in brackets and the overall data are unbracketed.

	nACE‐IPP	nACE‐VPP
Crystallographic statistics		
Resolution (Å)	79.45–1.60	75.27–1.90
Space group	P1	P1
Cell dimensions		
Lengths, a, b, c (Å)	74.23, 103.40, 115.42	73.34, 78.22, 83.01
Angles, α, β, γ (°)	84.95, 85.49 81.55	88.75, 64.70, 75.25
Molecules per asymmetric unit	4	2
Completeness	97.2 (92.2)	97.9 (96.6)
*R*_pim_	0.061 (0.859)	0.083 (1.29)
<I/σI>	6.4 (0.7)	7.2 (0.6)
CC1/2	0.998 (0.344)	0.997 (0.357)
Multiplicity	6.9 (6.0)	6.9 (6.1)
Refinement statistics		
*R*_work_/*R*_free_	0.18/0.21	0.19/0.23
RMSD bonds (Å)	0.02	0.01
RMSD angles (°)	1.93	2.05
Ramachandran angles		
Favoured %	98.51	98.59
Allowed %	1.28	1.25
Outliers %	0.21	0.17
Average B‐factors (Å^2^)		
Amino acids	22.55	40.76
Ions	16.79	31.62
Ligands	45.89	65.06
Water	31.52	39.56
IPP/VPP peptides	14.68	27.29
Number of non‐hydrogen atoms		
Amino acids	20 411	9924
Ions	12	5
Ligand	819	321
Water	2325	444
IPP/VPP peptides	92	44
PDB code	8QFX	8QHL

### The tripeptides IPP and VPP preferentially inhibit nACE

In a previous study, kinetic analysis of ACE inhibition by IPP and VPP performed on sACE from cell lysate did not account for domain selectivity [38]. To assess this, the IC_50_ values of IPP and VPP were determined by fixed‐time fluorometric assays using the substrate Z‐Phe‐His‐Leu with recombinantly isolated nACE and cACE. IPP and VPP were synthesised with the *trans* configuration and displayed lower IC_50_ values for nACE compared to cACE (Table 2), which indicates a slight preference for nACE (domain‐selectivity values of 12.10 and 18.59, for IPP and VPP respectively (Table 2)). Similar N‐selectivity for IPP and VPP was revealed by Lunow et al. using tripeptides isolated from fermented milk, which is thought to contain a mixture of *cis/trans* configurations of these peptides [12]. Based on previous molecular docking simulations [38, 51], both IPP and VPP were modelled as adopting a *cis* configuration between Ile/Val1 and Pro2 to facilitate binding, in which the terminal amino group interacts with His491 (cACE‐His513) instead. However, given that the *trans* configuration is four times more favoured than the *cis* configuration in proline‐containing peptide bonds [52], our experimental findings support that, the *trans* configuration may be preferred, in which the deprotonated terminal amino group of the Ile1/Val1 residue, along with its carbonyl group, straddles the zinc ion as observed in both nACE‐IPP and nACE‐VPP crystal structures. However, co‐crystallisation of nACE in complex with the tripeptides isolated from milk could be carried out to further investigate the isomer selectivity.

**Table 2:** Potency and selectivity of IPP and VPP tripeptides towards sACE, nACE and cACE. The IC_50_ values for sACE were determined previously using the Hip‐His‐Leu substrate [32]. Errors are shown as ± SEM.

Tripeptide	IC_50_ (μm)	
	IPP	VPP
sACE	5.00	9.00
nACE	1.42 ± 0.24	2.65 ± 0.09
cACE	17.18 ± 0.90	49.28 ± 4.04
Selectivity factor^a^	12.10	18.70

^a^ IC_50_(C)/IC_50_(N).

### Comparison of nACE‐IPP and cACE

**Fig. 3:**
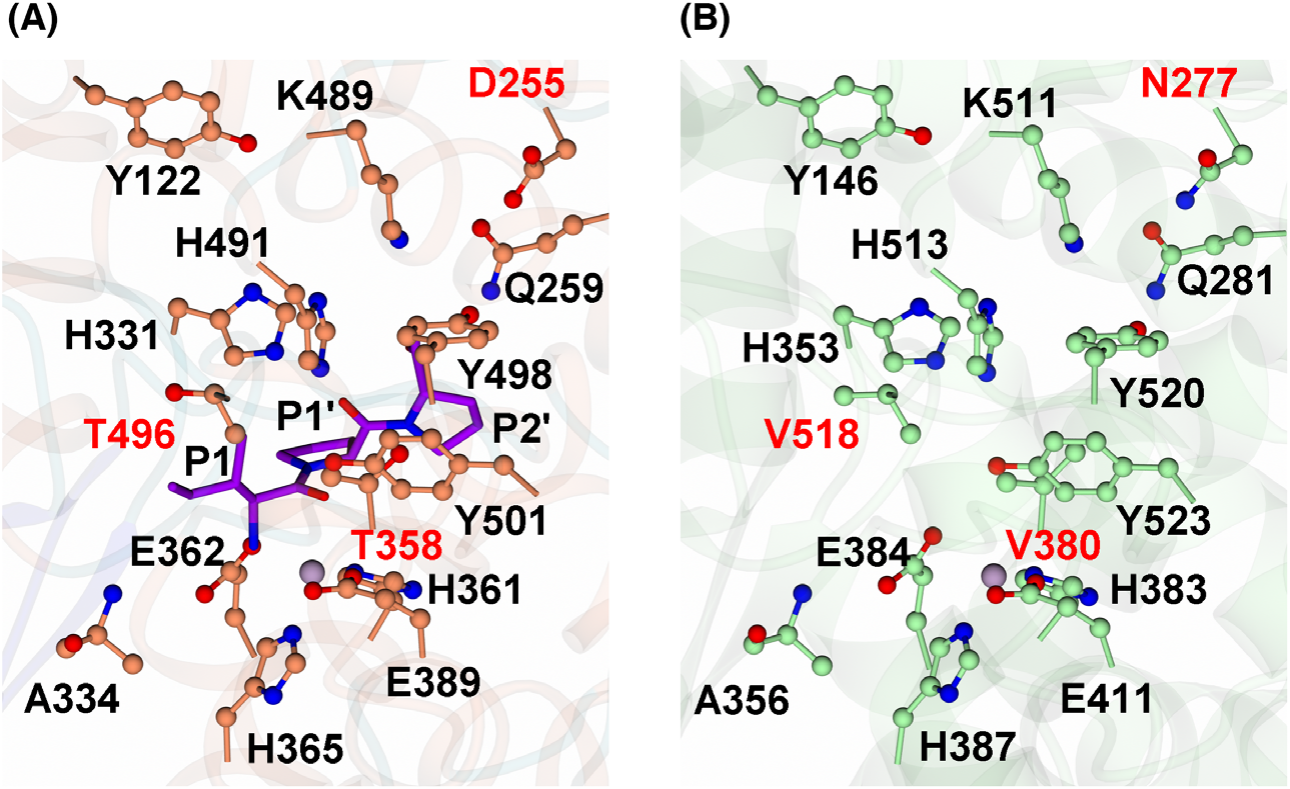
nACE and cACE active site residues. (A) nACE‐IPP peptide bound in the active site. (B) Equivalent cACE active site residues for comparison. nACE residues are shown in orange, cACE residues in green, IPP in purple and zinc ions in lilac. Non‐equivalent residues in the active site are highlighted by red text. Figure was generated using CCP4MG.

Attempts at co‐crystallising cACE with IPP or VPP proved unsuccessful. Therefore, to identify potential structural features that may result in the increased specificity of the tripeptides IPP and VPP for nACE, the previously determined minimally glycosylated cACE structure (PDB code 1O8A) was compared with the nACE‐IPP structure determined in the present study (Fig. 3A,B). At the active site, cACE and nACE display a near perfect alignment across the residues (RMSD of 0.92 Å for 560 Cα atoms of molecule A), with no differences in the position of interacting residues, suggesting that cACE will bind IPP and VPP in a similar conformation. With respect to the S_1_ subsite, there is only a single different amino acid residue between nACE (Thr496) and cACE (Val518). This increased hydrophobicity within the S_1_ subsite for cACE suggests that tighter binding of the P_1_ amino acids (Ile/Val) within cACE would occur. The effect of an increase in hydrophobicity within the S_1_ subsite is further evidenced by the decrease in IC_50_ values of IPP relative to VPP for both cACE and nACE, where the addition of a single carbon atom (VPP to IPP) decreases the IC_50_ to 2.87 and 1.87‐fold respectively. However, given that IPP and VPP have increased binding to nACE, the S_1_ subsite does not significantly contribute to domain specificity for these tripeptides. All residues within the S_1_′ subsite (for nACE‐IPP) are conserved in cACE, as well as the residues involved in direct hydrophobic and hydrogen bonding interactions within the S_2_′ subsite. However, there are differences in the extended network of water‐mediated interactions from the terminal carboxyl group, which may contribute to the enhanced specificity towards nACE. Differences at residues nACE‐Asp255 (cACE‐Asn277) and Thr358 (Val380), which both interact with the P_2_′ proline through two bridging waters, would account for weaker binding to cACE, as cACE‐Val380 is incapable of forming a hydrogen bond, and cACE‐Asn277 will likely form a weaker interaction than nACE‐Asp255. Additionally, previous work that elucidated the nACE ‘open’ structure [50] and that of the nACE‐specific inhibitors 33RE and RXP407, has revealed that residues distal from the direct‐bonding residues are also involved in domain specificity. Interestingly, it was revealed that S_2_′ subsite mutations of nACE to their cACE counterpart completely abolished affinity for 33RE [53]. The S_2_′ nACE residues (Ser260, Glu262, Asp354, Ser357, Thr358 and Glu431) were shown to encourage subdomain closure for 33RE [54] and RXP407 [39] by providing a more polar subdomain interface than cACE, which would allow further water‐mediated interactions to stabilise the tripeptides. Therefore, the subtle differences in specificity of IPP and VPP towards nACE could be driven by the S_2_′ subsite residues involved in subdomain closure. Although IPP and VPP may bind to cACE in a similar orientation, the complex may be less thermodynamically stabilised despite a more hydrophobic environment at the S_1_ subsite.

### IPP/VPP tripeptide binding mode in comparison to the nACE‐specific inhibitors 33RE and RXP407

To further analyse the subtle nACE‐selective inhibition by IPP and VPP, we superimposed nACE‐IPP with nACE‐33RE (PDB code 4BXK [54]) and nACE‐RXP407 (PDB code 3NXQ [39]). The interactions at the S_1_′ and S_2_′ position are conserved, with potentially stronger binding at these sites for IPP and VPP peptides due to the inclusion of stronger hydrophobic contacts from the proline rings. However, RXP407 and 33RE are significantly more selective for nACE (*K*_*i*_(C)/*K*_*i*_(N)) over cACE than IPP or VPP (RXP407 = 2896, 33RE = 927 [55, 56]. This is due to better contacts at the S_1_ and S_2_ subsites, as IPP and VPP peptides lack large functional groups that extend into the S_2_ subsite, while RXP407 and 33RE are able to make nACE‐specific contacts with Tyr369 (cACE‐Phe391) and Arg381 (cACE‐Glu403). These findings further support that nACE selectivity is driven by both direct contacts at the S_1_ and S_2_ subsites, and by indirect contacts distal from the S_2_′ subsite. The distal interactions are involved in subdomain closure around the substrate, as well as encouraging a larger network of water‐mediated interactions in comparison to cACE [50, 53].

### Comparison of nACE‐IPP to the bradykinin potentiating peptide (BPPb) and N‐domain‐specific peptide (Ac‐SDKP)

Bradykinin potentiating peptides (BPPa,b,c and 2) contain the ‘IPP’ sequence at their C terminus and many are shown to be C‐domain‐specific inhibitors [55]. Previously reported crystal structures of nACE and cACE in complex with BPPb revealed the mechanism of binding [56, 57]. In comparison to the nACE‐IPP peptide structure, the P_1_′ and P_2_′ prolines of BPPb occupy identical positions within nACE and cACE, however the extended N terminus alters the geometry with respect to the P_1_ position of the IPP peptide such that it does not form a coordinating bond with the catalytic zinc ion in the BPPb‐bound structures. Together, these results further support the hypothesis that IPP and VPP peptides will display the same orientation within cACE, as described with the crystal structures of nACE‐IPP and nACE‐VPP in the present study, with the P_2_′ position possessing strong preference for proline with a terminal carboxyl. Additionally, the crystal structure of nACE in complex with the dipeptide KP from the cleavage of the natural nACE selective substrate, Ac‐SDKP, indicates that the position Pro3 is identical to that of the cleaved C‐terminal end of its substrate. The IPP tripeptide may therefore mimic the cleaved product at this site.

**Fig. 4:**
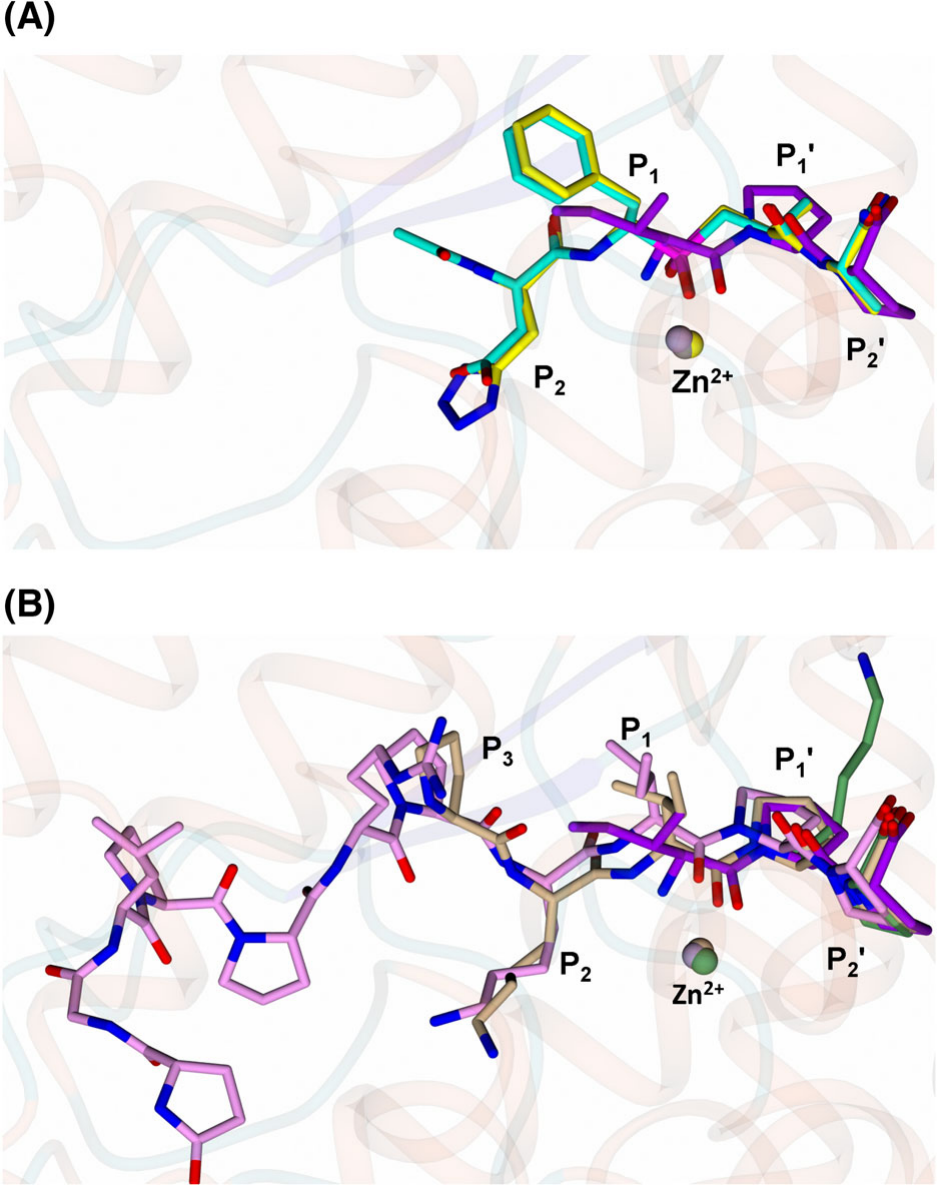
nACE‐IPP peptide complex in comparison. (A) Comparison to nACE specific inhibitors 33RE and RXP407 and (B) Comparison to bradykinin potentiating peptide (from nACE‐BPPb and cACE‐BPPb structures) and Ac‐SDKP peptide (hydrolysis dipeptide product KP shown). IPP is shown in purple, 33RE in yellow, RXP407 in cyan, BPPb in beige (nACE) and pink (cACE) and Ac‐SDKP dipeptide KP in dark green. Figure was generated using CCP4MG.

Due to the previously observed displacement of the zinc ion by BPPb in cACE, the low occupancy in nACE, and the conserved ‘IPP’ in BPPs (Fig. 4B), we performed occupancy refinement of all zinc ions within the asymmetric unit of nACE‐IPP peptide complex structure (with 4 molecules in the asymmetric unit) and nACE‐VPP (with 2 molecules in the asymmetric unit) peptide complex by iteratively refining the occupancies of each zinc ion. Based on the presence of positive mFo‐Fc electron density at occupancies lower than 1, we concluded that zinc is not displaced by the IPP or VPP peptides, and that the IPP/VPP motif of BPPb is not what drives zinc displacement as observed previously [45, 46].

## Conclusion

Interactions at the S_2_ subsite are important for domain selectivity [1], but require the presence of large functional groups that extend into this subsite to specifically target residues that vary between nACE and cACE. This can affect the bioavailability of potent nACE‐ and cACE‐selective inhibitors which, despite their clear selective inhibition, make poor drug candidates for targeted anti‐fibrotic/inflammatory and anti‐hypertension therapies respectively [5, 39, 58]. The high‐resolution crystal structures of nACE in complex with IPP and VPP presented here revealed the precise molecular interactions that facilitate binding of these ACE‐inhibitive tripeptides, whereby the terminal amino and carbonyl groups interact with the zinc ion, along with several hydrogen bonds and hydrophobic interactions that extend into the S_1_, S_1_′ and S_2_′ subsites. Based on the initial structural analysis of the interacting residues, one might predict cACE to be a better IPP/VPP binder due to the increased hydrophobicity within the S_1_ subsite which accommodates Ile/Val. Despite this, the kinetic data presented here show that *trans*‐IPP and *trans*‐VPP bind both nACE and cACE, but with selectivity towards nACE. Since all residues that form direct interactions with IPP/VPP in nACE are conserved in cACE [with the exception of Thr 496 (cACE‐Val 518)], and the IPP/VPP in nACE is similar to the C terminus of BPPb in terms of binding, cACE binding to IPP and VPP is likely to adopt a similar conformation to that observed in nACE‐IPP/VPP (presented here) and cACE‐BPPb (based on a previous study [57]). The subtle enhanced specificity towards nACE may, therefore, be due to both an increase in water‐mediated interactions and polarity within and adjacent to the S_2_′ subsite. This is further supported by previous mutagenesis work, which revealed that the increased polarity of nACE within this region is more conducive to subdomain closure than in the equivalent location within cACE [50, 59].

The tripeptides used in this study were obtained by chemical synthesis, as opposed to isolation from fermented milk or milk casein‐hydrolysates and were synthesised in the *trans* configuration. A comparison of the selectivity factors calculated previously [12] and in the present study suggest that nACE preferentially binds the *trans* configuration, and the nature of the interaction at the active site, with the terminal amino group and carboxyl group straddling the catalytic zinc, supports this hypothesis.

## Author contributions

KSG wrote the manuscript, performed all crystallographic analysis, analysed the data and edited the manuscript. GEC performed crystallisation, collected X‐ray diffraction data, analysed the data and edited the manuscript. SLUS performed the expression and purification of nACE and cACE and the kinetic characterisation of IPP and VPP binding. EDS analysed the data and edited the manuscript. KRA supervised the study, analysed the data and edited the manuscript. All authors reviewed the manuscript.

### Peer review

The peer review history for this article is available at https://www.webofscience.com/api/gateway/wos/peer‐review/10.1002/1873‐3468.14768.

## Data Availability

The structural data that support these findings are openly available in the wwPDB at https://doi.org/10.2210/pdb under the accession codes 8QFX and 8QHL.
